# Assessment of water source availability and quality for small ruminant consumption in the Northern Badia region of Jordan

**DOI:** 10.14202/vetworld.2020.1073-1082

**Published:** 2020-06-12

**Authors:** J. Al-Khaza’leh, A. Abdelqader, M. Abuajamieh, F. M. F. Hayajneh

**Affiliations:** 1 Department of Nutrition and Food Processing, Faculty of Agricultural Technology, Al-Balqa Applied University, P.O. Box 19117, Al-Salt, Jordan; 2 Department of Animal Production, School of Agriculture, The University of Jordan, Amman 11942, Jordan

**Keywords:** Jordan, livestock, water access, water quality, water scarcity

## Abstract

**Background and Aim:**

Water is the most important nutrient for the production of healthy livestock. Water scarcity bottlenecks livestock production in arid and semi-arid regions, particularly during the dry season. This study aimed to assess water availability and quality for small ruminants, and to identify major challenges of meeting their water requirements in two major small ruminant production systems in Jordan.

**Materials and Methods:**

Transhumant and sedentary production systems in the Northern Badia region of Jordan were the focus of this study. A questionnaire was distributed to 120 sheep and goat farmers (62 transhumant farmers and 58 sedentary farmers) and a water quality assessment was completed.

**Results:**

Results showed that the two production systems varied their water source use seasonally. Water provision was perceived as one of the major constraints for Bedouins, particularly during the dry season in transhumant production systems, when longer distances to water sources and the high costs of fetching water daily aggravated the problem. The mean distance and travel times to the boreholes were less in the sedentary system. Watering frequency was significantly lower in the transhumant system compared to the sedentary system (p<0.05). Although the values of water quality parameters complied with guidelines for livestock consumption, low water quality was the main concern expressed by some of the survey households.

**Conclusion:**

Technical support to properly design, manage, and rehabilitate surface water harvesting systems is required for the sustainable use of water resources in the study region. Moreover, systematic water quality monitoring is necessary to ensure its suitability for livestock use. Further investigations on the microbiological quality of water and its effect on the health and performance of livestock are recommended.

## Introduction

The success of Jordanian livestock farming systems depends on small ruminant animals, which represents the largest proportion of biomass in the country [1]. These animals are the primary livelihood of Jordanian farmers living in rural and marginal regions, as they contribute to food security and risk assurance [2]. Despite adaptive traits that enable these animals to thrive in arid and semi-arid environments, small ruminant livestock productivity is often limited by various factors, such as feed and water shortages, diseases, and rangeland reductions [3]. The increased temperatures and declining amounts of precipitation associated with climate change in Jordan have amplified these limiting factors (especially water shortages) [4].

Livestock productivity and health are dependent on the quantity and quality of water consumed, given that water is a vital nutrient that contributes to an animal’s body composition, growth, reproduction, and biological processes [5]. Any restriction on normal water intake adversely affects livestock productivity and health [5]. According to Deutsch *et al*. [6], around 10% of the annual global water flow is used by the livestock sector. About 87.5% of that 10% is used to irrigate livestock feed crops, while the small remaining fraction (12.5%) is consumed or used for livestock servicing and processing [5,7,8]. Although this percentage of water used for livestock consumption is small in comparison to water used for feed crop production [9], drinking water availability is extremely important in arid and semi-arid regions, where water and pasture quality and availability are low, and environmental temperatures are high. Daily drinking water requirements range from about 2 to 12 L per head [10], although this amount is species and breed-dependent. Water intake requirements are also influenced by other internal and external factors, such as body weight, physiological state, physical activity, productivity, ration composition, water quality, and environmental conditions [8]. Water quality is also vitally important, as poor water quality can negatively affect livestock health or reduce its palatability for drinking, thereby affecting its consumption [11].

Jordan is considered one of the water-poorest countries in the world [12]. Water scarcity is a common problem; the increased refugee influx started in 2011 caused the per capita share of water to drop to 123 m^3^ per year [13]. The increasing population and limited water supply are expected to further decrease the per capita water supply to only 91 m^3^ per year within the next 5 years [14]. Water availability is also influenced by its high spatiotemporal variability within Jordan and its seasonal fluctuations (wet and dry seasons). Water quality is also a problem, which has deteriorated due to increased salinity and water pollution from agricultural, industrial, and domestic sources [14,15]. The Northern Badia region of Jordan is particularly affected by water shortages and poor water quality, and also suffers from additional factors due to the influx of Syrian refugees into the region [13].

We hypothesize that variation in seasonal environmental conditions, such as rainfall and temperature, could affect water’s availability and quality, and could also influence forage quality and production. We also hypothesize that such variations could have negative consequences on livestock productivity and health. Moreover, we think that variations in the management techniques (e.g., utilization and accessibility of drinking water) of two common farming systems used in the region could affect animal growth and production.

Studies addressing the availability, suitability, and utilization of seasonal drinking water in small ruminant production are limited, particularly in the Northern Badia region of Jordan. The present study, therefore, aims to assess the availability and quality of drinking water sources used for small ruminants and the associated challenges of meeting their water requirements in this region.

## Materials and Methods

### Ethical approval and Informed consent

This study did not involve the use or contact with live animals, and hence, ethical approval was not necessary. Informed consent was obtained from each participant.

### Study area

**Figure-1:**
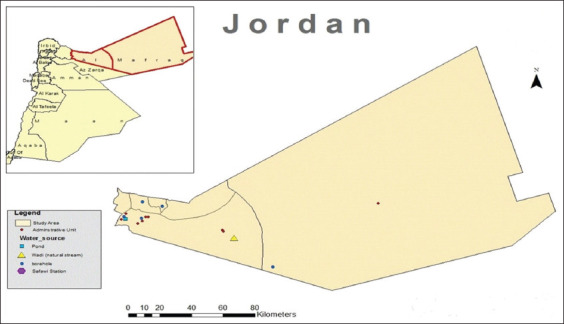
Location of the study area and sites of sampled water sources.

The study was carried out in the Northern Badia region of Jordan, east of Mafraq governorate (Figure-1). The term “Badia” refers to a desert or arid region where Bedouins or Badu dwell. The Northern Badia region constitutes 36% of the total area of Badia (km^2^ = 71,474), which itself constitutes 80% of the total area of Jordan. The region is predominantly inhabited by local Bedouins, and Syrian refugees living in host communities and camps, in particular the Al-Zaatari camp. The region was selected due to its arid climatic conditions to assess the water availability and quality challenges faced by small ruminant production systems. Farmers in this region use either a transhumant or sedentary (agro-pastoral) livestock production system. Transhumant production systems are pastoral, with herders who practice two major patterns of seasonal mobility: Eastward “al tashreeg” to benefit from grazing during late winter and early spring, then back westward “al taghreeb” after that. Northern Badia has an arid climate and has been affected by frequent droughts cycles, receiving a total annual rainfall of about 116 mm. Aridisol is the dominant soil type in the study region [16] and is rich in limestone and basalt stones. Water and pasture biomass availability in the region is limited and water quality commonly deteriorates due to agriculture and domestic runoff, and the consistent overuse of groundwater, which leads to declining water tables and increased salinity levels.

The predominant species used in the small ruminant production systems in Northern Badia are sheep and goats. According to secondary data obtained from the study region’s agricultural offices in 2017, the livestock population in the study area is estimated to be about 366,940 head of sheep, 61,210 head of goats, 3020 head of cattle, and 1130 head of camels. The same data showed that in 2017, the total population of sheep and goat keepers was 3150. For the same year, sheep and goat populations in the study area constituted of 12% and 7.9% of the total population of sheep (n=3,063,120) and goats (n=772,670) in Jordan, respectively [17].

### Sampling, study period and data collection

After the study site was selected, a list of all small ruminant farmers and their livestock holdings were obtained from local officials from the Ministry of Agriculture for each village in the study region. Our list of farmers included those who kept at least ten adult animals in their flock. From this list, 120 farmers were selected using systematic random sampling by taking every third name. A single-visit survey was conducted from the beginning of December 2017 to the end of March 2018 using a structured questionnaire format. The questionnaire’s suitability regarding its language and logical flow was confirmed by locals before conducting the survey. The final sample included 62 transhumant production farms and 58 sedentary farms.

The questionnaires focused on socio-economic characteristics of each household, any water problems (quantity and quality), and any potential related challenges regarding farming practices and seasonal water shortages. In addition to the survey, secondary data on the number of small ruminants and small ruminant keepers, price of water, and the status of water sources intended primarily for livestock were obtained from water and agricultural offices in study area. Climate data were acquired from the Meteorological Department of Jordan (Amman, Jordan).

### Water sampling and analysis

The names of drinking water sources for small ruminants were listed with the help of each local community and agricultural office. Water samples were collected in the rainy (February-March) and dry (July-August) seasons of 2018 and were selected to adequately cover the quality of the major water sources intended for small ruminants in Northern Badia. In both sampling periods, a total of 13 water samples were collected from different water sources: Five boreholes, one rain harvesting pond, and one wadi (natural stream). For the rain harvesting pond, the analysis was performed only during the first sampling period (rainy season) because it dried up during the dry season. A composite sample was collected at each site with consideration given to each site’s spatial and temporal features. A composite sample was taken at three different locations at three different times at the rain harvesting pond, as it is a stagnant water source. The other sites were all running water sources (borehole and wadi); therefore, composite samples were taken at regular time intervals.

Chemical parameters, namely, electrical conductivity (EC), total dissolved solids (TDS; characterized by carbonates, chlorides, sulfates, nitrates, sodium, potassium, calcium, and magnesium), and the pH of each water source were analyzed during both sampling periods. These parameters were intentionally selected because their high levels can adversely affect water and feed intake in livestock, thus affecting animal productivity [5]. Salinity was also assessed, which was more likely to occur from the increased extraction of groundwater [13]. EC, TDS, pH, and temperature were recorded *in situ* using a portable meter (HI-991300, Hanna^®^).

### Statistical analysis

Each household’s characteristics and perceptions regarding seasonal water availability, quality, and utilization were expressed in means, percentages and frequencies. The Chi-square test for the proportions of categorical variables and the t-test for continuous variables were applied after checking the normal distribution of residuals, to compare the two production systems. The water variable cost was not normally distributed; hence, the Wilcoxon-Mann–Whitney test was employed to detect any significant differences between transhumant and sedentary systems. For the suitability assessment, the quality parameters of water sources were compared with guideline values for livestock consumption [5]. The mean rank for each water source in each production system was calculated as

Σ(x_1_w_1_ + x_2_w_2_ + x_3_w_3_…x_n_w_n_)/total response count

Where x is the response count (frequency) for each water source, and w is the weight of ranked position (the higher the mean rank, and the better the quality of the water source). All statistical analyses were performed using SAS software version 9.3 (SAS Institute Inc, Cary, NC, USA).

## Results

### Household characteristics

The household survey data are summarized in Table-1. All interviewed household heads were male, with an average of 36 years of experience in livestock husbandry, and an average family size of ten. The majority of the respondents (77.6%) in the sedentary system depend on family labor for the herding and caring of their animals, while the transhumant systems (45.2%) had more continuous or seasonal labor. The illiteracy level among the household heads was higher in the transhumant system (51.6%). Household heads using the sedentary system were higher educated (75.9%).

**Table-1:** General characteristics of households in two production systems.

Variables	Transhumant (n=62)			Sedentary (n=58)			p-value
		
Continuous variables	n	Mean	SD	n	Mean	SD	
Household size	60	10.5	6.0	58	9.5	5.6	0.3399
Experience (years)	60	36.2	13.1	58	35.3	16.4	0.7623
Number of livestock by species							
Goats	25	74.4	61.1	39	51.3	44.8	0.0861
Sheep	60	468.2^a^	471.7	47	169.7^b^	209.1	0.0001
Total herd size	62	483.1^a^	467.7	58	172.0^b^	194.5	0.0001

**Categorical variables**	**n**	**(%)**		**n**	**(%)**		

Male household head (%)	62	(100)		58	(100)		
Education of household head (%)			^-^				
Illiterate	32	(51.6)		14	(24.1)		0.0024
Literate	30	(48.4)		44	(75.9)		0.0024
Labor source (%)							
Family labor	34	(54.8)		45	(77.6)		0.0097
Hired labor	28	(45.2)		13	(22.4)		0.0097

SD=Standard deviation of mean, N=Number of respondents, Means in the same row with different superscript letters differ significantly at p<0.05 (t-test for means or Chi-square for proportions)

Sheep and goats were the most often kept small ruminant animals; 37% (transhumant) and 48% (sedentary) of the flocks were mixed species of sheep and goats. Sheep were the most dominant livestock species owned by the majority of farmers. The well-known Awassi breed was the primary sheep breed in the study area kept by all farmers (100%). A few farmers (5%) also kept Naemi and Najdi sheep breeds in addition to the Awassi breed. The northern desert goat was the primary goat breed (47%), followed by Mountain Black (35%) and crossbreeds (18%). More farmers raised sheep in the transhumant system than in the sedentary system. This difference was significant (p<0.05), but only a marginal difference was observed for goat flock size. Flock sizes were larger in the transhumant system than in the sedentary system (p<0.05; Table-1).

### Seasonal water sources for small ruminants use

**Figure-2:**
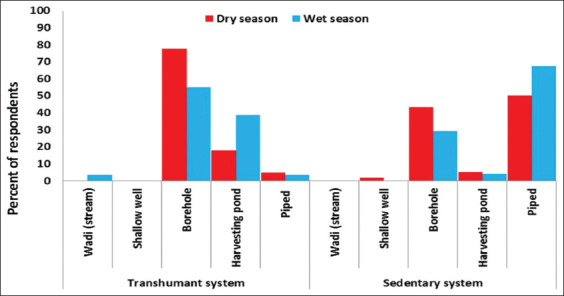
Water sources for small ruminants during the dry and wet seasons in the transhumant and sedentary systems.

Figure-2 displays the different water sources that were used in each system for small ruminants. Ground water (boreholes) or surface water (streams and water harvesting ponds [dugouts]) sources were used in both systems. High seasonal variation was observed in the utilization of the various water sources. In the transhumant system, 77% (dry season) and 55% (wet season) of the respondents (n=62) used boreholes as the major source of drinking water, followed by rain harvesting ponds during the dry and wet seasons (18% vs. 39%). In the sedentary system (n=58), 50% (dry season) and 67% (wet season) used piped water (tap water) as the main source water source, followed by boreholes (Figure-2).

**Figure-3:**
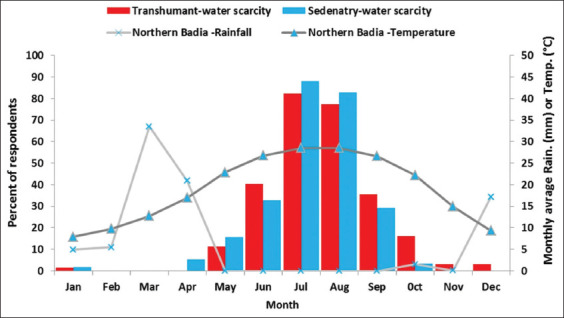
Monthly diagram of water scarcity in the study area in relation to average monthly rainfall and temperature distribution covering the years 2016-2017 (data were obtained from the Jordanian Meteorological Department for Safawi station).

Water scarcity by month is depicted in Figure-3. There was large variation between the dry (April-October) and wet (November-March) seasons in the farmers’ perception of water scarcity. The majority of respondents in both production systems experienced water scarcity during the dry season, which coincides with the typical changes to rainfall amounts and ambient temperature patterns (Figure-3).

### Seasonal water frequency and accessibility for small ruminant use

Table-2 displays the differences in watering frequency between the two groups by season. In general, animals were watered frequently. The lowest watering frequency occurred in the transhumant system during the dry season. According to survey respondents, some households watered their flocks once a day (11.3%), twice a day (28.2%), thrice a day (5.6%), or freely (54.9%) in the transhumant system, compared with 2.6%, 13.8%, 6.9%, and 76.7% in the sedentary system, respectively. None of the surveyed households in the transhumant and sedentary system watered their flocks once a day during the dry season or thrice a day during the wet season. The majority of animals were watered freely (65.0% vs. 65.8%), followed by twice a day (22.5% vs. 22.0%) in dry and wet seasons, respectively. The proportion of respondents who water their flock freely was significantly lower (p<0.05) in the transhumant system than in the sedentary system, while the proportion of respondents who water their flock once and twice a day in the transhumant system was significantly higher (p<0.05) than the corresponding frequencies in the sedentary system.

**Table-2:** Watering frequency of small ruminants in the study area by production system and season.

Frequencies	Production system		p-value*	Season		p-value*
	
	Transhumant	Sedentary		Dry season	Wet season	
			
	n (%)	n (%)		n (%)	n (%)	
Once a day	14 (11.3)	3 (2.6)	0.0159	0 (0.0)	17 (14.2)	0.0012
Twice a day	35 (28.2)	16 (13.8)	0.0073	27 (22.5)	24 (20.0)	0.6361
Thrice a day	7 (5.6)	8 (6.9)	0.6895	15 (12.5)	0 (0.0)	0.0015
Free access	68 (54.9)	89 (76.7)	0.0004	78 (65.0)	79 (65.8)	0.8921
Total	124 (100)	116 (100)	0.0001	120 (100)	120 (100)	0.2873

N=Number of respondents,

* Statistically significant between production system and season at p<0.05 (Chi-square test)

As shown in Table-3, the average distance traveled by transhumant farmers from their homesteads to the boreholes during the dry season averaged 21.3 km. The trip, including the time walking to the borehole and waiting at the watering point, took an average of 122.8 minutes. During the wet season, distance traveled was 18.9 km and time spent on the task was 119.0 min. In contrast, distance traveled in the sedentary system was 9.2 km (dry season) and 10.4 km (wet season), with an average time of 57.6 min (dry season) and 73.5 min (wet season). The mean distance and time needed to the boreholes were significantly higher (p<0.05) during the dry season in the transhumant system than during the dry season in the sedentary system. In the transhumant system, the mean distance to reach the rain harvesting water source tended to be non-significantly higher (p=0.0830) in the dry season than in the wet season. The differences between seasons within each production system were not significant (p>0.05).

**Table-3:** One-way mean trip and travel time needed to access the water sources by season in the transhumant and sedentary system.

Production system	Water source	Distance (km)				Time needed (min)			
	
		Dry		Wet		Dry		Wet	
			
		n	Mean±SD	n	Mean±SD	n	Mean±SD	n	Mean±SD
Transhumant (n=62)	Wadi (stream)	0	-	2	1.0±0.0	0	-	2	17.5±3.5
	Shallow well	0	-	0	-	0	-	0	-
	Borehole	48	21.3^a^±25.0	34	18.9±22.6	48	122.8^a^±85.2	34	119.0±114.6
	Harvesting pond	11	43.6±29.3	24	25.5±27.3	11	158.2±87.0	24	108.1±88.0
	Piped	3	0.0±0.0	2	0.0±0.0	3	0.0±0.0	2	0.0±0.0
Sedentary (n=58)	Wadi (stream)	0	-	0	-	0	-	0	-
	Shallow well	1	2.0	0	-	1	30.0	0	-
	Borehole	25	9.2^b^±8.5	17	10.4±8.4	25	57.6^b^±48.2	17	73.5±45.8
	Harvesting pond	3	31.7±7.6	2	20.0±28.3	3	110.0±62.4	2	45.0±63.6
	Piped	29	0.0±0.0	39	0.0±0.0	29	0.0±0.0	39	0.0±0.0

^c^For boreholes the means in transhumant and sedentary system with different superscripts are significantly different (p<0.05); n: number of respondents; SD=Standard deviation

### Cost of water used for small ruminant consumption

Water cost (water *per se* and transportation) used for small ruminant consumption in the study area ranged from 0 to 3000 Jordanian Dinar (JD) per year (1 JD ≈ 1.4 USD in 2017) and was dependent on flock size, water source, production system, time of year, distance from water sources, connection to the water network, quantity used, and water quality. For instance, waters from natural streams and rain harvesting ponds were used free of cost by a considerable proportion of farmers, 14% in the sedentary and 50% in the transhumant system. Water itself, without its transportation cost, will probably constitute the smallest portion of the total variable costs in both systems. Farms connected to the water network pay a subsidized price per cubic meter based on the amount of water consumed. Farms not connected to the water network have to purchase water from a supplier per cubic meter. Due to the fact that the water price is subsidized in the study area and accessible to most farmers for free, the problem of water accessibility was reduced. There was no significant difference in the cost of water between production systems with overall median and mean of 428.8 and 37.5 JD in the transhumant system, and 266.2 and 120.0 JD in the sedentary system, respectively.

### Seasonal measured water quality for small ruminants use

Table-4 represents the chemical quality of water sources used for small ruminant consumption. All chemical parameters of water intended for livestock consumption in both sampling periods complied with livestock consumption guidelines. The values of EC and TDS were higher during the dry season than during the wet season. pH values were similar between the two sampling periods (Table-4). No statistically significant difference was revealed between the parameters’ mean values in either season.

**Table-4:** Water quality parameters of water sources during the rainy and dry seasons in comparison to guidelines values for livestock consumption.

Sampling period	Parameters	n	LSM±SE	Min	Max	Guidelines^c^	Compliance
Rainy season	EC (us/cm)	7	653.1±154.8	303	1252	na	-
	TDS (mg/L)	7	417.9±99.1	194	801	3000	Yes
	pH (unit)	7	7.6±0.2	7.3	8.3	6-8.5	Yes
Dry season	EC (us/cm)	6	697.5±167.2	352	1307	na	-
	TDS (mg/L)	6	446.1±107.1	225	837	3000	Yes
	pH (unit)	6	7.6±0.2	6.8	8.0	6-8.5	Yes

n=Number of water sources measured; SE=Standard error; na=Not available;

c The upper maximum levels are concentrations above which problems could occur in livestock (Beede, 2012)

### Herders’ perception on water source quality

**Figure-4:**
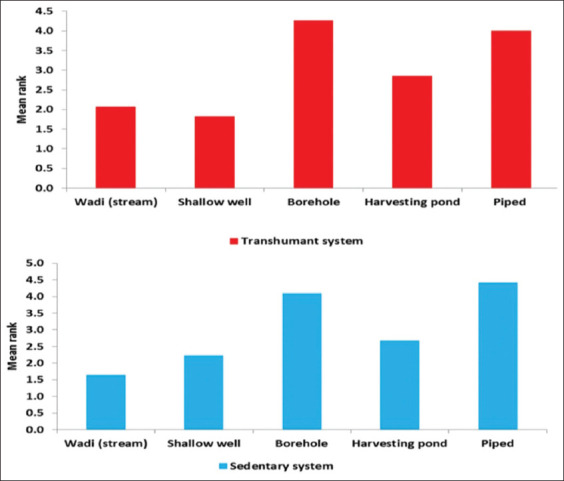
Comparison of water sources used for animal consumption in transhumant (top panel), and sedentary (bottom panel) by their quality (The higher the mean rank, the better the quality of the water source).

A comparison of the major water sources used based on their quality is displayed in Figure-4. The top-ranked water source with good quality in the transhumant system was the borehole, followed by piped water, rain harvested pond water, and wadi. In the sedentary system, the top-ranked water source with good quality was piped water, followed by the borehole, rain-harvested pond water, and shallow wells. Shallow wells and wadi water sources in the transhumant and sedentary systems, respectively, were perceived to be of low quality. However, a small number of respondents, 1.7% in the sedentary and 3.3% in the transhumant systems, reported occurrences of deaths or diseases in their flock due to poor water quality.

**Figure-5:**
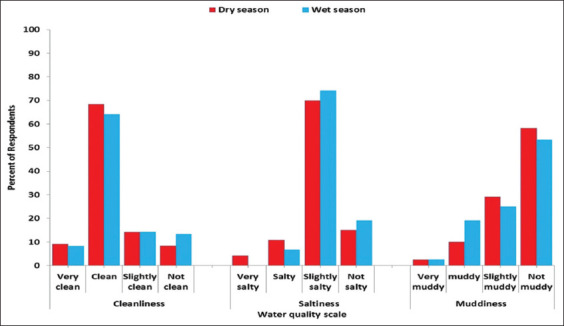
Farmers’ perception on water quality: Cleanliness; saltiness; and muddiness by season.

Water quality, as perceived by farmers in both seasons, is presented in Figure-5. Most of the respondents perceived water quality as clean (68% and 64%). The majority of respondents perceived water to be slightly salty during the dry (70%) and wet (74%) seasons. A considerable proportion of the respondents perceived water quality as non-muddy (58% and 53%). Some perceived the water as slightly muddy in the dry season (29%) and the wet season (25%).

## Discussion

In Jordan, the adoption of one farming system over the other by farmers is largely influenced by cultural, socio-economic, and environmental factors. Moreover, the decision of farmers to adopt different farming systems can also be affected by demographic characteristics, the costs and benefits of production, and their adaptive capacity to environmental conditions. Small ruminant production in the Northern Badia region of Jordan improves the resilience and livelihood of herders. In the present study, a large number of goats and sheep in the transhumant (74.4 vs. 468.2) and sedentary (51.3 vs. 169.7) systems kept by farmers is linked with their tolerance and adaptive capacity to the arid and semi-arid environments. Farmers who own a mixture of species and breeds may be better able to utilize the animals’ different ecological niches and vegetation [18], which improves complementary economic benefits and counteracts any climate variability impacts [19-21]. Availability of water resources and their water quality is crucial for livestock production in arid and semi-arid regions and plays a determinant role in the sustainability of livestock and farmer mobility in the study area.

Groundwater (boreholes) is the primary water source in Jordan, and the only vital water source in some regions [15]. In the transhumant production system, boreholes and rain harvesting ponds were the major water sources used for livestock consumption. This result is consistent with the findings of Al-Tabini *et al*. [22]. In the sedentary system, the lower proportion of farmers using rain harvesting ponds could be ascribed to their high dependence on the pipe-borne water supply schemes. According to the community and agricultural offices, the problem of seasonal water shortages was counteracted by the construction of “hafa’ir” (surface water harvesting dugouts) to collect the water during the wet season, or drilling boreholes. However, water supply for livestock was a serious problem during the dry season, because most surface water sources dried up or were severely deteriorated. Our results are in line with Al-Khaza’leh *et al*. [23] and Al-Assaf [24], who listed water shortage as one of the primary constraints on small ruminant production in Jordan.

In our study, watering frequency of livestock varied considerably across season and production system. The higher observed watering intervals in the dry season compared to the wet season could be ascribed to the prevailing high ambient temperatures during the dry season and low moisture content of the feed. In arid and semi-arid area, where high ambient temperatures are prevalent and the feasibility of providing shade materials difficult, thermal stress on grazing animals can be expected [25]. Long exposures to thermal stress along with water shortages, low forage water content, and feed quality adversely affect animal productivity [25]. Another study by Thornton *et al*. [26] showed that temperature increases have a negative effect on pastoral livestock production by indirectly impacting pasture productivity, water availability, and disease prevalence. Frequent free access to drinking water in the transhumant system was more difficult than in the sedentary system, due to the number of households directly connected to the water network in the sedentary system.

Although the majority of transhumant and sedentary farmers encountered water shortages, particularly during the dry season, livestock watering frequency was apparently not affected. Measuring the actual amount of drinking water consumed by livestock in extensive production systems is difficult. Meeting the water consumption needs of a mixed species and breed herd at any one water interval is also difficult. The watering frequency in this study is consistent with the findings of Wurzinger *et al*. [27], who reported that watering intervals during the summer and winter seasons for Syrian Jabali Mountain goats were twice and once a day, respectively. Similar observations were reported by Beyene *et al*. [28], who indicated that watering frequency of small ruminants in Southern Ethiopia was higher in the dry season than in wet season. A higher watering interval in the wet season compared to the dry season was reported for goats in Ethiopia, however [29]. Watering frequency of small ruminants in this study was shorter compared to other species in other reports in Ethiopia [30,31] which found that camels were the highest tolerant species with the longest watering interval (15 days), followed by small ruminants (4-5 days) and cattle (2-3 days).

This study showed that water accessibility in terms of distance traveled and time spent on watering tasks varied between the two production systems, seasons and water sources. Most farmers lived further away from water sources, and only 4.8% of transhumant farmers had access to water through the pipe network, while 50% of sedentary farmers had access. In the present study, transhumant farmers travel such long distances to watering points in comparison to sedentary farmers due to differences in their adaptability to environmental changes and the pastoral nature of transhumant farmers.

Respondents also stated that the connection of households to water pipe networks in the study area did not necessarily ensure constant water accessibility. Moreover, due to the high number of farmers utilizing the few sparse water sources, farmers traveled long distances and waited for long periods at the water sources for their turn to fill their water tanks. This was more time-consuming and uneconomical, and leads to competition and conflicts among pastoralists. A study by Tolera and Abebe [31] showed that during the dry season and in times of drought, pastoralists in Southern Ethiopia walked long distances (up to 5 h) to access water. According to Al-Tabini *et al*. [22], daily fetching of water from the wells in the Badia region of Jordan was difficult and uneconomical for the Bedouins (sometimes driving a two-way trip of up to 60 km length). Therefore, the cost of water transport from its sources should be considered an important variable in livestock production in the region.

With reference to the measured water quality results of this study, all chemical parameters were within the permissible limit for livestock consumption. The lower water levels in the dry season caused the chemical parameters to be higher than in the wet season. There were, however, a few boreholes with water suitable for livestock but not human consumption, based on secondary data acquired from agricultural offices in the study area. This was primarily due to the high level of other elements in these boreholes, such as sulfate content. According to Beede [5], livestock can tolerate water with sulfates up to 300 mg/l before their health suffers.

Water source preference with regard to quality has been seen from different perspectives. Farmers in both production systems attributed the low quality of surface water sources (wadi, shallow well and rain harvesting pond) compared to piped and borehole water sources, to be due to potential microbial and chemical contaminations by human and animal activities surrounding the sources, because they are unprotected. From another perspective, farmers ascribed the high quality of boreholes and piped water due to the disinfection of piped water against pathogens, and due to the protection of these sources from potential contamination.

## Conclusion

The present study showed that the supply of drinking water was perceived as one of the major challenges for Bedouins in the Northern Badia region of Jordan. The seasonal variation in water availability and accessibility is a challenge for small ruminant production in the study area, particularly in the transhumant production system. This study confirms the previous findings of water shortage problem which was associated with long travel times and distances, as well as the high cost incurred from the need to fetch water daily.

Due to various variables, such as human population growth, industrial and agricultural expansion, regional geological features, mismanagement, overuse and depletion of groundwater resources, the availability and quality of water sources in the study area are expected to be adversely impacted in the coming years. Moreover, climate change, particularly increasing temperatures, and frequent droughts are likely to exacerbate water scarcity and contribute to declining water quality, and to accentuate the impacts of the aforementioned factors.

Technical support in the suitable design, rehabilitation and proper management of surface water sources are necessary to improve conditions in the study area. Water sources should be established as close to homesteads and grazing area as possible to alleviate the farmers’ water provision burdens. Although, the values of water quality parameters did not exceed the guidelines for livestock consumption, low-water quality was considered problematic by some of the surveyed households. Frequent monitoring of the water quality could ensure its suitability for livestock consumption. Further investigations on the microbiological quality of water and its effect on the health and performance of livestock are recommended.

## Authors’ Contributions

JA was in charge of designing and conducting the study, collecting and analyzing the data and samples, interpreting results, and writing of the manuscript. AA, MA, and FMFH participated in writing, interpreting, and editing the manuscript. All authors read and approved the final manuscript.
